# Impact of immediate breast reconstruction on perioperative therapy: insights from a Japanese Nationwide Registry

**DOI:** 10.1007/s12282-024-01604-3

**Published:** 2024-06-19

**Authors:** Shinsuke Sasada, Hiraku Kumamaru, Naoki Hayashi, Naoko Kinukawa, Masakazu Toi, Hiromitsu Jinno, Shigehira Saji

**Affiliations:** 1 Department of Surgical Oncology, Research Institute for Radiation Biology and Medicine, Hiroshima University, 1-2-3 Kasumi, Minami-ku, Hiroshima City, Hiroshima 734-8551 Japan; 2 Department of Healthcare Quality Assessment, University of Tokyo, 7-3-1 Hongo, Bunkyo-ku, Tokyo, 113-8655 Japan; 3 Division of Breast Surgical Oncology, Department of Surgery, Showa University School of Medicine, 1-5-8 Hatanodai, Shinagawa-ku, Tokyo, 142-8666 Japan; 4 Department of Breast Surgery, Tokyo Metropolitan Cancer and Infectious Diseases Center, Komagome Hospital, 3-18-22 Honkomagome, Bunkyo-ku, Tokyo, 113-0021 Japan; 5 Department of Surgery, Teikyo University School of Medicine, 2-11-1 Kaga, Itabashi, Tokyo 173-8606 Japan; 6 Department of Medical Oncology, Fukushima Medical University, 1 Hikarigaoka Fukushima, Fukushima, 960-1295 Japan

**Keywords:** Breast cancer, Immediate breast reconstruction, Mastectomy, Perioperative treatment, Rradiotherapy

## Abstract

**Background:**

Immediate breast reconstruction (IBR) is a common oncoplastic procedure used in breast cancer surgery. This study aims to investigate compliance with prosthetic breast reconstruction guidelines and its impact on perioperative treatment.

**Methods:**

We reviewed data from the National Clinical Database-Breast Cancer Registry between January 2019 and December 2020. We compared perioperative treatment implementation between the IBR and non-IBR groups by subtype matching for age, menopausal status, T stage, N stage, and histology.

**Results:**

A total of 8,860 patients with breast cancer who underwent IBR (6,075 breast prostheses, 2,492 autologous tissues, and 293 others) were identified. The compliance rate with the guidelines for prosthetic breast reconstruction was 97.7%. After matching, chemotherapy for luminal A-like diseases was significantly less frequent in the IBR group than in the non-IBR group (16.3% vs 20.5%, *p* < 0.001), and radiotherapy was less frequent in luminal A-like and HER2-positive patients (7.2% vs 9.0%, *p* = 0.010 and 7.1% vs 11.4%, *p* = 0.005, respectively). Among the 1–3 node-positive cases, fewer patients with prosthetic IBR received radiotherapy than those without IBR (15.7% vs 26.4%, *p* < 0.001).

**Conclusion:**

Prosthetic breast reconstruction was performed with strict adherence to the Japanese guidelines. The implementation rates of chemotherapy and radiotherapy were lower in the specific IBR group than those in the non-IBR group. Therefore, large-scale, long-term follow-up data are required.

**Supplementary Information:**

The online version contains supplementary material available at 10.1007/s12282-024-01604-3.

## Introduction

Breast cancer is the most common cancer among adult women worldwide and in Japan. The number of newly diagnosed breast cancer cases in Japan has increased from 85,856 in 2013 to 97,142 in 2019 [1]. Mastectomy is a standard radical surgery, along with breast-conserving surgery. However, it has severe cosmetic disadvantages. Immediate breast reconstruction (IBR) is an important treatment option for this demerit [2]. The National Insurance Service of Japan covered IBR using breast prostheses in 2013, and breast reconstruction increased dramatically, from 1,050 in 2013 to 5,942 in 2018 [3]. The prosthetic IBR fell to 3,678 in 2019 due to an interruption in the supply of prosthetic reconstructive materials based on concerns of breast implant-associated anaplastic large-cell lymphoma, but has since increased again, reaching 4,587 in 2021.

However, the oncological safety of IBR for locally advanced breast cancer has not been adequately studied [4]. Therefore, the Japan Oncoplastic Surgery Society recommends the following requirements for using breast prostheses: 1) stage II or lower preoperative diagnosis, 2) no evidence of skin or pectoralis major muscle involvement, and advanced lymph node metastasis [3]. However, compliance with these recommendations has not been investigated. In addition, there is concern that IBR may influence the implementation of standard adjuvant therapy. IBR is associated with a higher risk of postoperative complications, such as wound complications, infections, flap necrosis, and implant removal [5, 6]. Prolonged recovery from complications can delay or prevent subsequent therapy. In particular, radiation has been reported to increase IBR-related complications and cause a reduction in cosmetic appearance and patient satisfaction [7, 8].

Given these gaps, our study aims to shed light on the impact of IBR on oncological outcomes and treatment compliance. The National Clinical Database-Breast Cancer Registry (NCD-BCR) covers surgeries performed in almost all surgical facilities in Japan. Detailed data on IBR were collected from the registry in 2019. This study was conducted to investigate compliance with the IBR guidelines and the implementation of standard perioperative therapy in Japanese patients with breast cancer who underwent IBR.

## Materials and methods

### Patients

**Fig. 1:**
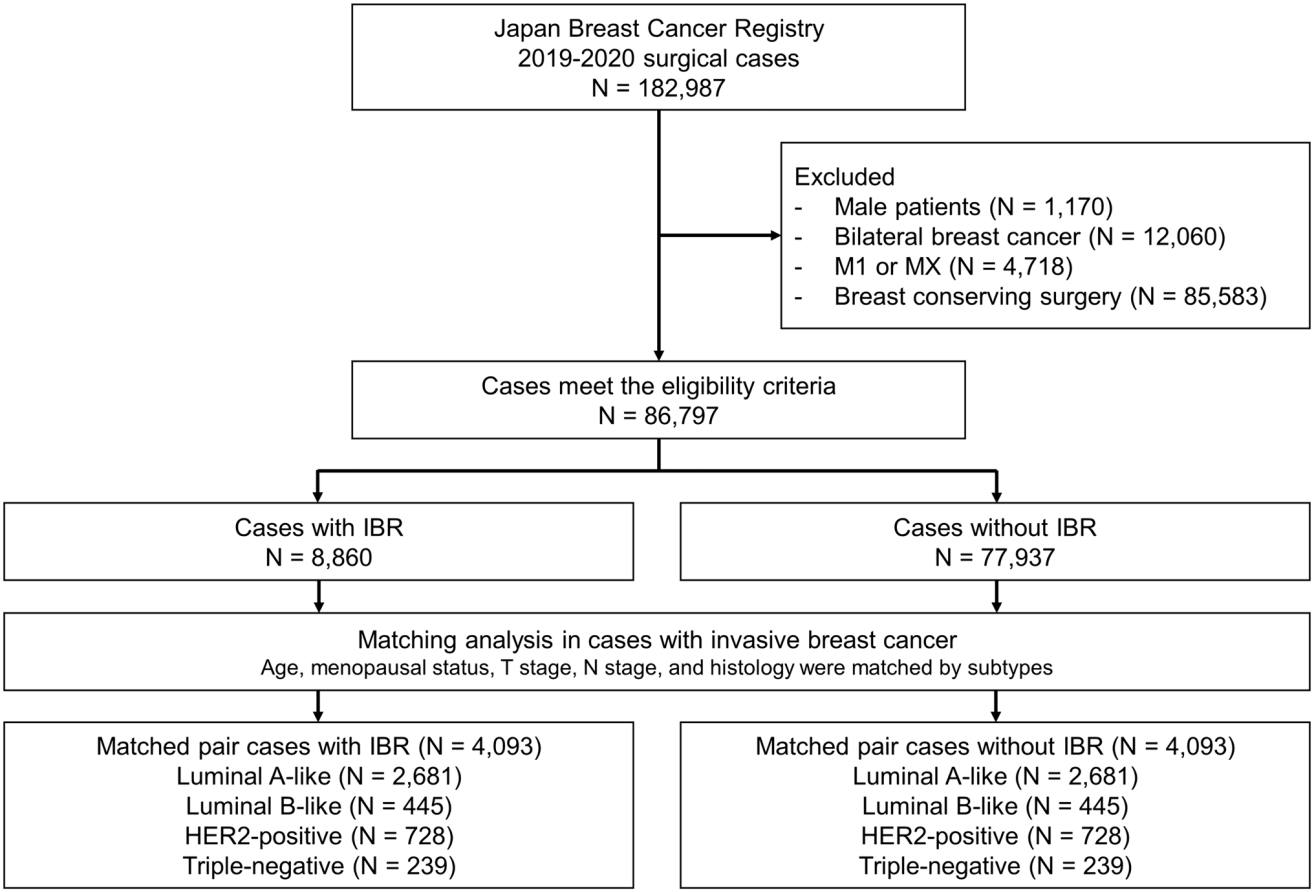
Study flowchart

Surgical cases with breast cancer registered in the NCD-BCR database between January 2019 and December 2020 were reviewed. The exclusion criteria were male sex, bilateral breast cancer, stage IV disease, and a history of breast-conserving surgery. A total of 86,797 patients were eligible for inclusion (8860 who underwent IBR and 77,937 who did not undergo IBR). We matched the IBR and non-IBR cases in a 1:1 ratio in terms of age, menopausal status, T stage, N stage, and histology by subtype to compare the implementation of perioperative therapy between the two groups. A flowchart of the process is shown in Fig. 1.

Subtypes were evaluated based on hormone receptors (HR), including estrogen receptor (ER), progesterone receptor (PgR), and human epidermal growth factor receptor type2 (HER2), and classified as luminal (HR-positive and HER2-negative), HER2-positive (HR-positive or HR-negative and HER2-positive), and triple-negative (HR-negative and HER2-negative). Luminal breast cancer was classified as luminal A-like (nuclear grade 1–2 and Ki-67 labeling index < 30%) or luminal B-like (nuclear grade 3 or Ki-67 labeling index ≥ 30%).

### Statistical analysis

All statistical analyses were performed using SAS ver.9.4 (SAS Institute, Cary, NC, USA). Statistical comparisons of categorical variables were performed using the Chi-square or the Fisher exact probability tests. Continuous variables were compared using the Wilcoxon rank-sum test. Continuous variables are expressed as medians and 5-95th percentiles. Differences were considered statistically significant at two-tailed *p* values < 0.05.

## Results

The characteristics of 8,860 patients with breast cancer who underwent IBR are presented in Table 1. Among them, 6,075 underwent IBR with breast prostheses, and 2,492 underwent autologous tissues. In the prosthetic and autologous IBR groups, 2.1% and 4.9% of the patients had T3–4 tumors, 5.9% and 10.4% had nodal metastases, and 5.0% and 9.9% underwent neoadjuvant chemotherapy, respectively.

**Table 1:** Characteristics of breast cancer cases with immediate breast reconstruction

	IBR (*n* = 8860) *n* (%)	Prosthetic (*n* = 6075) *n* (%)	Autologous (*n* = 2492) *n* (%)	Other (*n* = 293) *n* (%)
Age (y), median (5–95%)	48 (35–66)	48 (34–67)	48 (36–66)	48 (34–68)
Menopausal status				
Premenopause	5905 (66.6)	4036 (66.4)	1649 (66.2)	220 (75.1)
Postmenopause	2671 (30.1)	1832 (30.2)	770 (30.9)	69 (23.5)
Unknown	284 (3.2)	207 (3.4)	73 (2.9)	4 (1.4)
BMI (kg/m^2^)	21.8 (18.0–29.0)	21.6 (17.7–28.8)	21.9 (18.0–29.7)	22.0 (17.6–29.7)
cT stage				
T0	36 (0.4)	30 (0.5)	5 (0.2)	1 (0.3)
Tis	2802 (31.6)	1916 (31.5)	780 (31.3)	106 (36.2)
T1	3423 (38.6)	2415 (39.8)	917 (36.8)	91 (31.1)
T2	2288 (25.8)	1560 (25.7)	654 (26.2)	74 (25.3)
T3	189 (2.1)	107 (1.8)	76 (3.0)	6 (2.0)
T4	78 (0.9)	17 (0.3)	47 (1.9)	14 (4.8)
Unknown	44 (0.5)	30 (0.5)	13 (0.5)	1 (0.3)
cN stage				
N0	8209 (92.7)	5713 (94.0)	2230 (89.5)	266 (90.8)
N1	546 (6.2)	306 (5.0)	218 (8.7)	22 (7.5)
N2	67 (0.8)	40 (0.7)	25 (1.0)	2 (0.7)
N3	31 (0.3)	11 (0.2)	18 (0.7)	2 (0.7)
Unknown	7 (0.1)	5 (0.1)	1 (0.1)	1 (0.3)
Histology				
Ductal carcinoma	7470 (84.3)	5118 (84.2)	2118 (85.0)	234 (79.9)
Lobular carcinoma	524 (5.9)	367 (6.0)	143 (5.7)	14 (4.8)
Others	866 (9.8)	590 (9.7)	231 (9.3)	45 (15.4)
Subtype				
Luminal A-like	3575 (40.3)	2426 (39.9)	1031 (41.4)	118 (40.3)
Luminal B-like	588 (6.6)	430 (7.1)	136 (5.5)	22 (7.5)
Luminal-unknown	1470 (16.6)	1004 (16.5)	423 (17.0)	43 (14.7)
HER2-positive	1292 (14.6)	880 (14.5)	364 (14.6)	48 (16.4)
Triple-negative	367 (4.1)	259 (4.3)	100 (4.0)	8 (2.7)
Unknown	1568 (17.7)	1076 (17.7)	438 (17.6)	54 (18.4)
Neoadjuvant chemotherapy				
No	8278 (93.4)	5765 (94.9)	2245 (90.1)	268 (91.5)
Yes	574 (6.5)	305 (5.0)	246 (9.9)	23 (7.8)
Unknown	8 (0.1)	5 (0.1)	1 (0.1)	2 (0.7)

*BMI* body mass index, *HER2* human epidermal growth factor receptor type2, *IBR* immediate breast reconstruction

### Compliance with guidelines for breast prosthesis use.

Among 6,075 cases with prosthetic IBR, 33 were not evaluable for compliance with the IBR guidelines for prosthetic breast reconstruction, and 5,906 (97.7%) were compliant (Table 2). The reasons for non-compliance were stage III disease in 94 cases, skin or pectoralis major muscle involvement in 82 cases, and advanced lymph node metastases in 60 cases (including duplicates). Forty-four of 136 noncompliant patients received neoadjuvant chemotherapy.

**Table 2:** Compliance of requirements criteria for artificial breast use

	*n* (%)
Compliance	5906 (97.7)
Non-compliance	136 (2.3)
Without neoadjuvant chemotherapy	92 (1.5)
With neoadjuvant chemotherapy	44 (0.7)
Reason of non-compliance^*^	
Stage III	94 (1.5)
Skin or pectoralis major muscle involvement	82 (1.3)
N2–3	60 (1.0)

^*^Including duplicates

### Implementation of perioperative treatment

The implementation of perioperative therapy in the matched cases of invasive breast cancer is shown in Table 3, and the characteristics of the matched cases are shown in Supplementary Table S1. Chemotherapy was administered frequently in luminal B-like, HER2-positive, and triple-negative subtypes, with no significant difference between cases with and without IBR. Fewer luminal A-like tumors with IBR received chemotherapy than those without non-IBR cases (16.3% vs 20.5%, *p* < 0.001). Radiotherapy was provided less frequently in the IBR group than in the non-IBR group for luminal A-like and HER2-positive subtypes (7.2% vs 9.0%, *p* = 0.010 and 7.1% vs 11.4%, *p* = 0.005, respectively). Radiotherapy rates were approximately 2% lower for luminal B-like and triple-negative subtypes in the IBR group. Endocrine and anti-HER2 therapies were administered similarly in both groups.

**Table 3:** Perioperative treatment according to subtypes in matched cases of invasive breast cancer

	Luminal A-like			Luminal B-like			HER2-positive			Triple-negative		
	Non-IBR (*n* = 2681) *n* (%)	IBR (*n* = 2681)*n* (%)	*p*	Non-IBR (*n* = 445)*n* (%)	IBR (*n* = 445)*n* (%)	*p*	Non-IBR (*n* = 728)*n* (%)	IBR (*n* = 728)*n* (%)	*p*	Non-IBR (*n* = 239)*n* (%)	IBR (*n* = 239)*n* (%)	*p*
Endocrine therapy	2441 (91.0)	2413 (90.0)	0.19	362 (81.3)	372 (83.6)	0.38	359 (49.3)	378 (51.9)	0.32			
Chemotherapy	550 (20.5)	437 (16.3)	< 0.001	278 (62.5)	277 (62.2)	0.94	594 (81.6)	589 (80.9)	0.74	214 (89.5)	206 (86.2)	0.26
Anti-HER2 therapy							588 (80.8)	571 (78.4)	0.27			
Radiotherapy	241 (9.0)	192 (7.2)	0.01	54 (12.1)	44 (9.9)	0.28	83 (11.4)	52 (7.1)	0.005	31 (13.0)	26 (10.9)	0.48

*HER2* human epidermal growth factor receptor type 2, *IBR* immediate breast reconstruction

Table 4 shows the radiotherapy implementation status in radiotherapy-recommended cases based on the Japanese Breast Cancer Society Clinical Practice Guideline, i.e., T3–4 or N1–3 [9]. Fewer patients who underwent prosthetic IBR received radiotherapy than those without IBR (21.2% vs 34.0%; *p* < 0.001). The breakdown was as follows: 12.8% vs 17.6% for T3–4N0 cases (*p* = 0.246), 15.7% vs 26.4% for TanyN1 (*p* < 0.001), and 54.3% vs 58.8% for TanyN2–3 (*p* = 0.242). There were no significant differences between the radiotherapy implementation rates in autologous or other IBR and non-IBR cases. Among the patients who received radiotherapy, the period from surgery to radiotherapy administration was longer in the IBR group than in the non-IBR group (cases without adjuvant chemotherapy: 69 days [31–202] vs 51 days [28–106], *p* < 0.001; cases with adjuvant chemotherapy: 222.5 days [48.5–323.5] vs 174 days [32–273], *p* < 0.001; Supplementary Table S2).

**Table 4:** Radiotherapy implementation rate in radiotherapy-recommended cases^*^

	Non-IBR		IBR					
			Prosthetic		Autologous		Other	
	*n*	Radiotherapy *n* (%)	*n*	Radiotherapy *n* (%)	*n*	Radiotherapy *n* (%)	*n*	Radiotherapy *n* (%)
All cases	28,375	9649 (34.0)	1163	246 (21.2)^‡^	605	217 (35.9)	63	28 (44.4)
T3–4N0^†^	2831	498 (17.6)	109	14 (12.8)	66	16 (24.2)	11	2 (18.2)
TanyN1^†^	17,486	4609 (26.4)	877	138 (15.7)^‡^	433	132 (30.5)	36	14 (38.9)
TanyN2–3^†^	7688	4522 (58.8)	173	94 (54.3)	101	69 (68.3)	14	12 (85.7)

*IBR* immediate breast reconstruction

^*^T3–4 or N1–3 cases based on Japanese Breast Cancer Clinical Practice Guideline

^†^Clinical stage if receiving neoadjuvant chemotherapy

^‡^*p* < 0.001 compared with non-IBR cases

## Discussion

We investigated the current status of IBR in breast cancer surgery in Japan and its impact on perioperative treatment using the NCD-BCR. Autologous IBR tend to be performed for more advanced diseases. Prosthetic IBR was performed according to the Japanese guidelines. Perioperative treatment, particularly radiotherapy for N1 cases and chemotherapy for luminal A-like diseases, was less common in the IBR group than in the non-IBR group.

Breast reconstruction after mastectomy is a common oncoplastic procedure for restoring breast symmetry and improving health-related quality of life (HRQoL) and psychological damage in survivors of breast cancer [2, 10]. IBR does not affect the prognosis or incidence of local recurrence after mastectomy [11]. However, in the Japanese Breast Cancer Society survey, 47.4% of physicians thought that IBR could mask local recurrence, and 27% thought it influenced prognosis [12, 13]. They were concerned about the delay or omission of adjuvant chemotherapy and radiotherapy.

 Radiotherapy is a significant risk factor for complications after breast reconstruction, particularly for breast prostheses [14]. In addition, radiotherapy impairs cosmetic outcomes, HRQoL, and patient satisfaction [7, 8, 15]. Therefore, the negative impact of IBR on the administration of postmastectomy radiation therapy is a concern. Postmastectomy radiation therapy is recommended for node-positive breast cancer to reduce both recurrence and mortality of breast cancer [16]. If the axillary lymph node is negative, radiotherapy is recommended for patients with T3–4 primary tumors [17]. The Japanese Breast Cancer Society Clinical Practice Guidelines also recommend postmastectomy radiation therapy for patients with T3–4 or positive-node disease [9]. No radiotherapy has reported as an independent risk factor for local recurrence in patients with IBR [18]. Recently, the therapeutic effect of postmastectomy radiation therapy for patients with 1–3 positive nodes has been discussed based on advances in modern systemic therapy [19, 20]. In the Japan Clinical Oncology Group survey, postmastectomy radiation therapy was performed in only 20.3% of T1–2N1 cases in 2016 [21]. Many physicians doubt the effectiveness of postmastectomy radiation therapy in patients with N1 disease. The recommendation for radiotherapy in T3–4N0 cases was not mentioned in the guidelines during the study period [22]. Regarding the timing of radiotherapy administration, the waiting period for radiotherapy was longer in IBR cases than in non-IBR cases. Although a delay of 8–20 weeks in the initiation of radiotherapy after breast-conserving surgery is associated with worsening local recurrence and survival, the relationship between the timing of radiotherapy after mastectomy with IBR and clinical outcomes is uncertain [23, 24].

In this study, the systemic therapy was similarly administrated to matched patients with and without IBR, excluding chemotherapy for luminal A-like invasive diseases. The indication for chemotherapy for luminal disease is based not only on clinical risk but also on multi-gene assays. Determining appropriate chemotherapy administration for the luminal A-like subtype is difficult, except in definitive high-risk cases. Particularly in premenopausal women, who account for the majority of patients with IBR, the potential effects of ovarian function suppression are unclear. The ongoing NRG-BR009 (OFSET) trial will provide insight into the need for chemotherapy in premenopausal women with ovarian function suppression. The risk–benefit assessment of chemotherapy in relation to IBR may affect treatment decision-making in patients with luminal A-like disease.

Previous meta-analyses have demonstrated that neoadjuvant chemotherapy did not increase IBR-related complications and that IBR did not delay the administration of adjuvant chemotherapy [25–27]. The Japanese Breast Cancer Society group survey demonstrated the oncologic safety of neoadjuvant chemotherapy in Japanese patients with IBR [28]. Recently, the de-escalation of local therapy for remarkable responders has been discussed. NSABP B-51/RTOG 1304 trial (NCT01872975) demonstrated the oncological safety of omitting chest and regional radiotherapy after mastectomy in cN + /ypN0 patients who received neoadjuvant chemotherapy [29]. The role of radiotherapy is changing with the development of systemic therapy, and IBR will be offered to more patients undergoing neoadjuvant chemotherapy in the near future.

This study has some limitations. First, this study was limited by its retrospective design. Second, the NCD-BCR had missing data, particularly regarding factors related to subtype classification. Third, this study had a short period for a database study, covering only 2 years. This is because data collection on IBR and subtype classifications began in 2019. However, this allowed the study to reflect recent Japanese trends and distinguish between luminal A- and B-like subtypes. Fourth, this study did not clarify the reasons for treatment administration or mention the relationship between IBR and the implementation of chemotherapy and radiotherapy. Despite the use of matching analysis to mitigate confounding factors, inherent selection biases may have persisted. Information about postoperative complications and HRQoL, which were not collected in the NCD-BCR database, may have affected treatment selection. The Japan Oncoplastic Breast Surgery Society group survey on HRQoL after IBR and postmastectomy radiation therapy is ongoing. Finally, this study lacks prognostic information.

Japanese physicians faithfully complied with the guidelines for prosthetic breast reconstruction and tended to perform autologous IBR in advanced cases. Perioperative treatment was administered less frequently in specific IBR cases, especially radiotherapy in N1 cases and chemotherapy in luminal A-like cases. IBR should be performed considering cosmetics, HRQoL, complications, and potential prognostic risks. In the future, more patients should be given opportunities for IBR, including those who have received neoadjuvant chemotherapy. Large-scale prognostic and HRQoL data from long-term follow-up are required.

### Supplementary Information

Below is the link to the electronic supplementary material.

Supplementary file1 (DOCX 24 KB)

## Data Availability

The data that support the findings of this study are not openly available due to the nature of the clinical data used. The clinical data are derived from the registry, which is not an open database. The data were accessed by a designated statistician through an application process approved by academic societies. Therefore, we are unable to offer the original clinical data.
