# Supplementary material for: Impact of immediate breast reconstruction on perioperative therapy: insights from a Japanese Nationwide Registry
Source: Breast Cancer. 2024 Jun 19;31(5):909–16. doi: 10.1007/s12282-024-01604-3 (PMC11341606; doi:10.1007/s12282-024-01604-3)
Supplement: Supplementary file 1 — Supplementary file1 (DOCX 24 KB) [file 12282_2024_1604_MOESM1_ESM.docx]

**Supplementary Table S1. Characteristics of matched cases of invasive breast cancer according to subtypes.**

|  | Luminal A-like | |  | | Luminal B-like | | | |  | | HER2-positive | | | |  | | Triple-negative | | | |
| --- | --- | --- | --- | --- | --- | --- | --- | --- | --- | --- | --- | --- | --- | --- | --- | --- | --- | --- | --- | --- |
|  | Non-IBR  (n = 2681)  n (%) | IBR  (n = 2681)  n (%) |  | Non-IBR  (n = 445)  n (%) | | IBR  (n = 445)  n (%) | |  | | Non-IBR  (n = 728)  n (%) | | IBR  (n = 728)  n (%) | |  | | Non-IBR  (n = 239)  n (%) | | IBR  (n = 239)  n (%) | |  |
| Age (y), median (5–95%) | 49 (35–66) | 49 (35–66) |  | 48 (37–67) | | 48 (37–67) | |  | | 47 (34–67) | | 47 (34–67) | |  | | 49 (33–68) | | 49 (33–68) | |  |
| Menopausal status |  |  |  | |  | |  | |  | |  | |  | |  | |  | |  | |
| Premenopause | 1873 (69.9) | 1873 (69.9) |  | | 315 (70.8) | | 315 (70.8) | |  | | 431 (59.2) | | 431 (59.2) | |  | | 131 (54.8) | | 131 (54.8) | |
| Postmenopause | 808 (30.1) | 808 (30.1) |  | | 130 (29.2) | | 130 (29.2) | |  | | 297 (40.8) | | 297 (40.8) | |  | | 108 (45.2) | | 108 (45.2) | |
| pT stage^*^ |  |  |  | |  | |  | |  | |  | |  | |  | |  | |  | |
| T1 | 1881 (70.2) | 1881 (70.2) |  | | 228 (51.2) | | 228 (51.2) | |  | | 422 (58.0) | | 422 (58.0) | |  | | 117 (49.0) | | 117 (49.0) | |
| T2 | 683 (25.5) | 683 (25.5) |  | | 198 (44.5) | | 198 (44.5) | |  | | 275 (37.8) | | 275 (37.8) | |  | | 100 (41.8) | | 100 (41.8) | |
| T3 | 107 (4.0) | 107 (4.0) |  | | 18 (4.0) | | 18 (4.0) | |  | | 22 (3.0) | | 22 (3.0) | |  | | 14 (5.9) | | 14 (5.9) | |
| T4 | 10 (0.4) | 10 (0.4) |  | | 1 (0.2) | | 1 (0.2) | |  | | 9 (1.2) | | 9 (1.2) | |  | | 8 (3.3) | | 8 (3.3) | |
| pN stage^*^ |  |  |  | |  | |  | |  | |  | |  | |  | |  | |  | |
| N0 | 2072 (77.3) | 2072 (77.3) |  | | 300 (67.4) | | 300 (67.4) | |  | | 533 (73.2) | | 533 (73.2) | |  | | 186 (77.8) | | 186 (77.8) | |
| N1 | 540 (20.1) | 540 (20.1) |  | | 124 (27.9) | | 124 (27.9) | |  | | 160 (22.0) | | 160 (22.0) | |  | | 44 (18.4) | | 44 (18.4) | |
| N2 | 58 (2.2) | 58 (2.2) |  | | 12 (2.7) | | 12 (2.7) | |  | | 28 (3.8) | | 28 (3.8) | |  | | 5 (2.1) | | 5 (2.1) | |
| N3 | 11 (0.4) | 11 (0.4) |  | | 9 (2.0) | | 9 (2.0) | |  | | 7 (1.0) | | 7 (1.0) | |  | | 4 (1.7) | | 4 (1.7) | |
| Histology |  |  |  | |  | |  | |  | |  | |  | |  | |  | |  | |
| Ductal carcinoma | 2403 (89.6) | 2403 (89.6) |  | | 441 (99.1) | | 441 (99.1) | |  | | 722 (99.2) | | 722 (99.2) | |  | | 236 (98.7) | | 236 (98.7) | |
| Lobular carcinoma | 278 (10.4) | 278 (10.4) |  | | 4 (0.9) | | 4 (0.9) | |  | | 6 (0.8) | | 6 (0.8) | |  | | 3 (1.3) | | 3 (1.3) | |

HER2, human epidermal growth factor receptor type2; IBR, immediate breast reconstruction.

^*^Clinical stage if receiving neoadjuvant chemotherapy.

**Supplementary Table S2. Period from surgery to radiotherapy in cases treated with radiotherapy.**

|  | Non-IBR | |  | IBR | | *p* |
| --- | --- | --- | --- | --- | --- | --- |
|  | n | Period (day)  median (5–95%) |  | n | Period (day)  median (5–95%) |  |
| All cases | 7746 | 63 (29–251) |  | 337 | 167 (36–315) | < 0.001 |
| Adjuvant chemotherapy |  |  |  |  |  |  |
| No | 4420 | 51 (28–106) |  | 137 | 69 (31–202) | < 0.001 |
| Yes | 3326 | 174 (32–273) |  | 200 | 222.5 (48.5–323.5) | < 0.001 |

IBR, immediate breast reconstruction.
